# Integrated transcriptomic and functional immunological approach for assessing the invasiveness of bivalve alien species

**DOI:** 10.1038/s41598-019-56421-y

**Published:** 2019-12-27

**Authors:** Alejandro Romero, Raquel Aranguren, Rebeca Moreira, Beatriz Novoa, Antonio Figueras

**Affiliations:** Instituto de Investigaciones Marinas (CSIC), Eduardo Cabello 6, 36208 Vigo, Spain

**Keywords:** Ecology, Immunology

## Abstract

Biological invasions started when humans moved species beyond their normal geographic limits. Bivalves are the most notoriously invasive species in subtidal aquatic environments. Next-generation sequencing technologies are applied to understand the molecular mechanisms involved in the invasion. The ecological immunology focuses on the role of immunity in invasion, and its magnitude could help to predict the invasiveness of alien species. A remarkable case of invasion has been reported in the Ría de Vigo (Spain) by the black pygmy mussel *Xenostrobus securis*. In Galicia, the Mediterranean mussel *Mytilus galloprovincialis* is the predominant cultured bivalve species. Can we predict the invasiveness of alien bivalve species by analyzing their immune response? Can *X. securis* represent a risk for the autochthonous mussel? We evaluated the suitability of the immune-related hypotheses in our model by using an integrated transcriptomic and functional immunological approach. Our analysis suggests lower immune capabilities in *X. securis* compared to *M. galloprovincialis*, probably due to the relocation of energetic resources from the immune response to vital physiological processes to cope with salinity stress. This multidisciplinary approach will help us understand how the immune response can be influenced by the adaptive process and how this immune response can influence the invasion process.

## Introduction

Biological invasions started when humans moved species beyond their normal geographic limits^1^. Most invasive species have negative impacts on the ecosystem and the economy of the affected area, with costs for controlling invasive species in the range of millions to billions of dollars per year^2^. Most studies in invasion ecology have been conducted in terrestrial plants^3^, but in recent years, marine and coastal ecosystems worldwide have been invaded at extraordinarily high rates due to shipping, aquaculture, live seafood trades, and climate change^4^. Although invasions in marine environments cover several taxonomic groups, bivalves are the most notoriously invasive species in intertidal/shallow subtidal rocky habitats^5^. Several aspects of the biology, ecology and evolution of non-indigenous bivalve species have been characterized to identify the key factors that could explain why some introduced species become invasive and others do not, to forecast their dissemination and to predict their impact on recipient communities^6^.

A recent area of investigation is ecological immunology, which focuses on the role of immunity in invasion ecology. The magnitude and strength of the immune response could help to predict the invasiveness of alien species^7^. Several immune-related ecological hypotheses may explain the success of invasive species. The enemy-release hypothesis (ERH) proposes that invaders are more competitive because they experience less pressure from natural enemies, including pathogens, than native species do. This allows the alien species to increase in abundance and distribution^8^. The “evolution of increased competitive ability (EICA) hypothesis” postulates that the invaders, in the absence of natural enemies, reallocate energetic resources from unnecessary defense mechanisms into fitness and growth^9^. However, this may lead the invasive species to be more vulnerable to re-infections and novel parasites^10^. The novel weapon hypothesis (NWH) suggests that invaders possess different or better defensive traits (e.g., novel biochemical molecules, higher amounts of antimicrobials) that potential enemies have never encountered before in their habitats^11^. Until now, empirical information on how native and invasive species respond to pathogens is very limited^10^.

Next-generation sequencing (NGS) technologies are being applied to understand the molecular mechanisms involved in the invasion process and to monitor and manage biological invasions^12,13^. Those studies resulted in the identification of several pathways responsible for the adaptation of the alien species to a broad range of physiological and biotic challenges when colonizing novel environments. For example, transcriptomic and proteomic studies applied to bivalves (e.g., *Mytilus galloprovincialis* and *Limnoperna fortunei*) revealed the importance of heat shock proteins in adaptation to thermal stress^14,15^. Only a few studies have paid attention to immunity, although it is known that innate immune system plays a role in the invasive European shore crab *Carcinus maenas*^16^ and an expanded family of antimicrobial peptides (AMPs) could help the harlequin ladybird (*Harmonia axyridis*) successfully outcompete native ladybirds^17^.

A remarkable case of invasion has been reported in the ecosystem of the Ría de Vigo (Galicia, Atlantic cost of Spain) by the black pygmy mussel *Xenostrobus securis*^18^. The Mediterranean mussel *M. galloprovincialis* is the predominant autochthonous bivalve species in Galicia, and its culture is a very important economic resource in the area. *X. securis*, endemic to New Zealand and Australian estuarine ecosystems, has spread throughout several Asian countries^19^ and the Mediterranean Sea^20^. Both *M. galloprovincialis* and *X. securis* are considered serious invasive species and are listed among the ‘100 worst invasive species’^21^. In Galicia, *X. securis* was first identified in the inner part of the Ría de Vigo and has spread via marine currents along the intertidal shores in this Ría and in other adjacent locations^22^. *X. securis* and the native *M. galloprovincialis* form patchy aggregations on muddy soft bottoms and intertidal hard rocks along the coastline of the inner Ría, where they compete for food and resources^23^. Because of the high production of *M. galloprovincialis* in Galicia, the true potential of the alien *X. securis* to colonize areas where Mediterranean mussel is established is an important issue that must be analyzed in detail.

Therefore, the aim of this work is to answer several questions: can we predict the invasiveness of alien bivalve species by analyzing their immune response and comparing it with that of an autochthonous species? Can the invader *X. securis* represent a serious risk for the autochthonous (but also invader) *M. galloprovincialis?* We evaluated the suitability of the immune-related hypotheses in our invasion model by using an integrated transcriptomic and functional immunological approach. Our analysis suggests lower immune capabilities in *X. securis* compared to *M. galloprovincialis*, probably due to the relocation of energetic resources from the immune response to vital physiological processes to cope with salinity stress. The transcriptomic and functional immunological data will help us understand how the immune response can be influenced by the adaptive process and how this immune response can influence the invasion process.

## Materials and Methods

### Animals and environmental conditions

**Figure 1:**
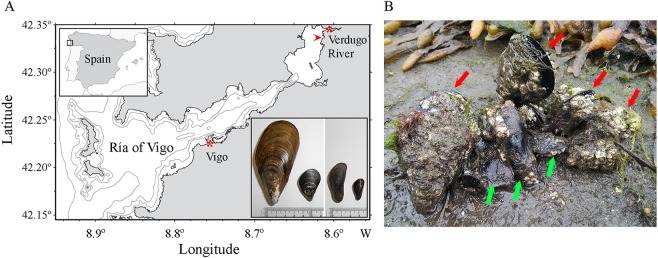
(**A**) The locations of sampling points at the Ría de Vigo (Spain) are indicated by asterisks. *X. securis* were only taken from the inner part of the Ría. *M. galloprovincialis* were only taken from the outer part of the Ría. Arrowhead indicates the third sampling point where animals were taken for histopathological analysis. *M. galloprovincialis* (insert, left) and *X. securis* (insert, right) are recognizable by their shape and size. (**B**) *X. securis* and the native *M. galloprovincialis* share the same habitat on muddy soft bottoms and intertidal hard rocks. *M. galloprovincialis* individuals (red arrows) are located in the upper part of the bunch, and *X. securis* individuals (green arrows) are at the bottom.

Animals were collected from three sites of the Ría de Vigo (NW, Spain) (Fig. 1). Two hundred adult black-pygmy mussels (*X. securis*, average shell length 42 ± 2.0 mm) were collected from the inner part of the Ría (42°20′47.5″N 8°36′24.1″W). In this location, the salinity ranges daily from 1.5 to 28 ppt due to the conjunction of the tide and the input of the fresh water from the river (*in situ* measurement). In this location *X. securis* have completely replaced the autochthonous mussel. For comparative purposes, the same number of juvenile *M. galloprovincialis* (average shell length 85 ± 15 mm) were collected from the outer part of the Ría (42°13′25″N, 8°45′37″W), where the water salinity is almost constant, ranging from 33 to 40 ppt^24^. Until now, the alien *X. securis* has not been detected in this area.

Animals were maintained at 15 °C in tanks with filtered sea water and fed daily with microalgae. The animals (*X. securis* and *M. galloprovincialis)* were divided into 2 groups; one group was maintained at a salinity of 23 ppt (conditions in the interior part of the Ría), and the other at a salinity of 34 ppt (conditions from the exterior part of the Ría) 30 days before the experiments. An intermediate sampling point at which both species coexist (42°19′29.8″N 8°36′53.2″) was selected to obtain samples for histological analysis. The salinity ranges in this point from 25 to 30 ppt (*in situ* measurement). At this location, 60 animals were collected and processed as described below.

### Sampling and sequencing

For transcriptomic analysis *X. securis* from the inner part of the Ría was sampled in summer. Small pieces of mantle were extracted from 6 animals and pooled for RNA extraction. Mantel tissue was selected for transcriptomic analysis to obtain a general overview of all the physiological processes activated under native environmental conditions since the transcriptome of immune related tissues such as hemolymph or gills is mainly enriched in immune processe^25^. Total RNA was isolated with the Maxwell 16 robot (Promega, Madison, USA). RNA purity and concentration were measured using a ND1000 spectrophotometer (NanoDrop Technologies, Inc., DE, USA). RNA integrity was tested on an Agilent Bioanalyzer 2100 system (Agilent Technologies, CA, USA). The RNA integrity value for the sample was 9.6. Next-generation sequencing was performed at Macrogen (Korea) following standard procedures. Sequencing was performed using Illumina HiSeq™ 4000 technology (Illumina, Inc., San Diego, CA, USA). For comparative purpose, the transcriptomic data of *M. galloprovincialis* previously obtained by Moreira *et al*.^25^ were used. In this case the mantle of 5 mussels from the outer part of the Ría were sampled in early summer and processed as previously described.

### Bioinformatic approach

CLC Genomics Workbench, v.10.0.1 was used to filter, assemble and perform the analyses of *X. securis* and *M. galloprovincialis*. Raw reads were trimmed to remove low-quality sequences, adaptors, and sequences shorter than 70 bp. Then, a transcriptome for each species was assembled with overlap criteria of 70% and a similarity of 0.9 to exclude paralogous sequences. The settings used were a mismatch cost = 2, deletion cost = 3, insert cost = 3, minimum contig length = 200 base pairs, and trimming quality score = 0.05. Next, RNA-seq analysis (mismatches = 2, length fraction = 0.8, similarity fraction = 0.8, and maximum hits per read = 10) was performed. The expression values were set as transcripts per million (TPM) and normalized by total transcript count and by average read length. Genes with a TPM value higher than 5 were used for the analysis. The contigs were identified in the UniProt/Swiss-Prot database using the Blast2GO software^26^ with an e-value threshold of 10e-5, and the GO terms were assigned. Then, enrichment analyses were conducted, including both mussel transcriptomes as the reference set. Fisher’s exact test was performed to find the over-represented biological processes for each species.

### Evaluation of immune-related gene expression by qPCR

Four immune-related genes were selected to confirm the transcriptomic expression values (TPM) by qPCR assays. The selected genes were C1q, MyD88, mytilin and myticin. Alignments of the selected sequences from the two species were conducted using MUSCLE software^27^ in order to confirm that the same orthologous genes were analyzed (Supplementary Fig 1). For the analysis, 24 × *. securis* and 24 *M. galloprovincialis* were collected from the inner and outer part of the Ría, respectively, and a fragment of the mantle was extracted. Six samples consisting of 4 pooled mantles were obtained from each species. Total RNA was extracted using the Maxwell 16 robot (Promega, Madison, USA). SuperScript II Reverse Transcriptase (Invitrogen) was used for the synthesis of the cDNA. Specific qPCR primers were designed using Primer3 software^28^, and their amplification efficiency was calculated by the Pfaffl method^29^ (Table 1). Quantitative PCR was performed on an MX3000 Thermocycler (Stratagene, Cedar Creek, TX, USA) using Brilliant II SYBR Green QPCR Master Mix (Agilent Technologies). The relative expression of each gene was normalized using elongation factor 1 alpha (EF1a) as a reference gene and was calculated using the Pfaffl method. Statistical comparisons of gene expression data between species were conducted using a T-test.

**Table 1:** Sequence of primers for the amplification of immune related genes by qPCR.

Name	Sequence	Efficiency
MYD88_Xen_For	GAGCCATCATTTTCCCAAGA	−3.0
MYD88_Xen_Rev	TTGATTTGCCCTTGTCCTTC	
MYD88_Myt_For	GCCACTATCAAGTGCCCTCT	−3.3
MYD88_Myt_Rev	TCTTCGGGTGGAAATTCTTG	
C1q64_Xen_For	CATGCTCCACAGGATTTGTC	−3.6
C1q64_Xen_Rev	TGGTCTGACTTTCCCTGCTT	
C1q64_Myt_For	CATGCTCCACTGGATTTGTC	−3.6
C1q64_Myt_Rev	TTGCATTTTCCATCCAGAGA	
Mytilin_Xen_For	ATGAGGCAGAGGCAAGTTGT	−2.6
Mytilin_Xen_Rev	TGCTCACTGGAACAACGAAG	
MytilinC_Myt_For	CCATGAAATTCTCCCCTGAA	−3.0
MytilinC_Myt_Rev	TCACCTTGTTCGGTTTCTCC	
MyticinC_Xen_For	CAGGAAGCCCAATCAGTAGC	−3.6
MyticinC_Xen_Rev	CGACAATGAAGGCAGTAGCA	
MyticinA_Myt_For	GCAGGTACGGAAGCTCATTC	−3.3
MyticinA_Myt_Rev	AAGGATTGTTCACCCTGCTG	
EFa1_Xen_For	CTCTCCGTCTCCCACTTCAG	−3.2
EFa1_Xen_Rev	TGGAGCAAAAACAACAACCA	
EFa1_Myt_For	CCACGAGTCTCTCCCAGAAG	−3.5
EFa1_Myt_Rev	TGCTGTCACCACAGACCATT	

### Characterization of *X. securis* hemocytes

The cell populations presented in the hemolymph of X*. securis* were characterized. Hemolymph was extracted from the adductor muscles of 15 adult *X. securis*, diluted 1:1 in cool filtered sea water (FSW) and individually analyzed in a FACSCalibur flow cytometer (BD Biosciences, San Jose, CA, USA). Density plots of relative size (FSC) and complexity (SSC) were constructed using data from one hundred and fifty thousand cells (Cell Quest software, BD Biosciences). For light microscopy, hemolymph cells were visualized in fresh preparations and in ethanol-fixed hemocytes after Hemacolor staining (Merck) using an ECLIPSE 80i microscope equipped with a Nomarski DIC prism (Nikon Corporation, Japan). The effect of water salinity was analyzed by flow cytometry. The cell distribution and number of hemocytes was measured in animals (N = 30) maintained in water at 23 ppt salinity or acclimated at 34 ppt for 30 days as previously described. In all experiments, hemolymph from adult *M. galloprovincialis* (N = 15) were also analyzed for comparative purposes.

### Phagocytic activity and reactive oxygen species (ROS) production of hemocytes

The ability of *X. securis* hemocytes to eliminate foreign particles by phagocytosis and ROS production was analyzed *in vitro*. The effect of water salinity was considered by using animals maintained at water salinities of 23 and 34 ppt. Three samples were generated by pooling hemolymph from 15X*. securis* and diluted in FSW. Similar samples from *M. galloprovincialis* were used for comparison. The cells were dispensed into 24-well plates (BD) and incubated for 30 min for adhesion. The non-adherent cells were removed by washing, and the remaining cells were incubated with different fluorescent particles at a 10:1 (particle:hemocyte) ratio: 1.0 μm FITC-Latex beads, *E. coli*–FITC and Zymosan A-FITC (Molecular Probes, OR, USA). After 3 h of incubation at 15 °C, un-internalized particles were removed, and the attached cells were mechanically resuspended in 500 ml FSW containing 0.03% trypan blue solution (Sigma). Then, 150,000 cells were analyzed in a FACSCalibur flow cytometer (BD Bioscience, San Jose, CA, USA). Cells were also stained with 3 μM DAPI solution (Invitrogen) and observed using a LEICA TCS SPE confocal microscope (Leica Microsystems, Germany). This experiment was repeated three times using animals maintained at 23 ppt salinity. The effect of water salinity and the kinetics of phagocytosis of Zymosan A-FITC were also evaluated using a similar experimental design. The percentage of cells that ingested Zymosan A-FITC particles was measured at 15, 30, 45, 60, 120 and 180 min after incubation. For ROS production, six samples consisting of pooled hemolymph from 6 animals were analyzed. Hemolymph was diluted in FSW, dispensed into 24-well plates, and the non-adhered cells were removed. The remaining cells were incubated for 30 min with 1 mg/ml Zymosan A (Sigma Aldrich). The control cells were treated with the same volume of FSW. After stimulation, the cells were washed and incubated for 10 min with 5 mg/ml of the 2′,7′-dichlorodihydrofluorescein diacetate probe (H2DCF-DA, Molecular Probes). The percentage of cells producing oxygen radicals was measured by flow cytometry. The median fluorescence index registered in the FL-1 channel was calculated as the ratio of stimulated samples to the control. All experiments were conducted three times. ANOVA followed by the Tukey test was carried out using GraphPad Prism 5 software (p < 0.005).

### Histopathology

Thirty invasive mussels and 30 autochthonous mussels were collected from the location where the two species coexist. Body sections (5 mm thick) were fixed in Davidson’s solution, embedded in paraffin and stained with Harris’ hematoxylin and eosin^30^. The presence and abundance of parasites was examined using an ECLIPSE 80i microscope (Nikon Corporation, Japan).

### Susceptibility of *X. securis* to *Vibrio splendidus* infection

Experimental infections were conducted to evaluate the susceptibility of *X. securis* at native (23 ppt) and introduced (34 ppt) salinity ranges to a bacterial infection. Three groups of 30 animals maintained at 23 ppt and the same number of animals maintained at 34 ppt were intramuscularly injected with 100 μl of a bacterial solution containing 5 × 10e8 CFUs/ml of the *Vibrio splendidus* LGP32 reference strain. This strain is pathogenic for *M. galloprovincialis*^31^. Additionally, 2 control groups of 30 animals were injected with the same volume of FSW. Mortalities were recorded 20 days after infection. For comparative purposes, the same number of *M. galloprovincialis* was infected. The results were plotted as Kaplan–Meier survival curves and analyzed with the log-rank (Mantel-Cox) test.

## Results

### Transcriptome analysis and functional annotation

**Figure 2:**
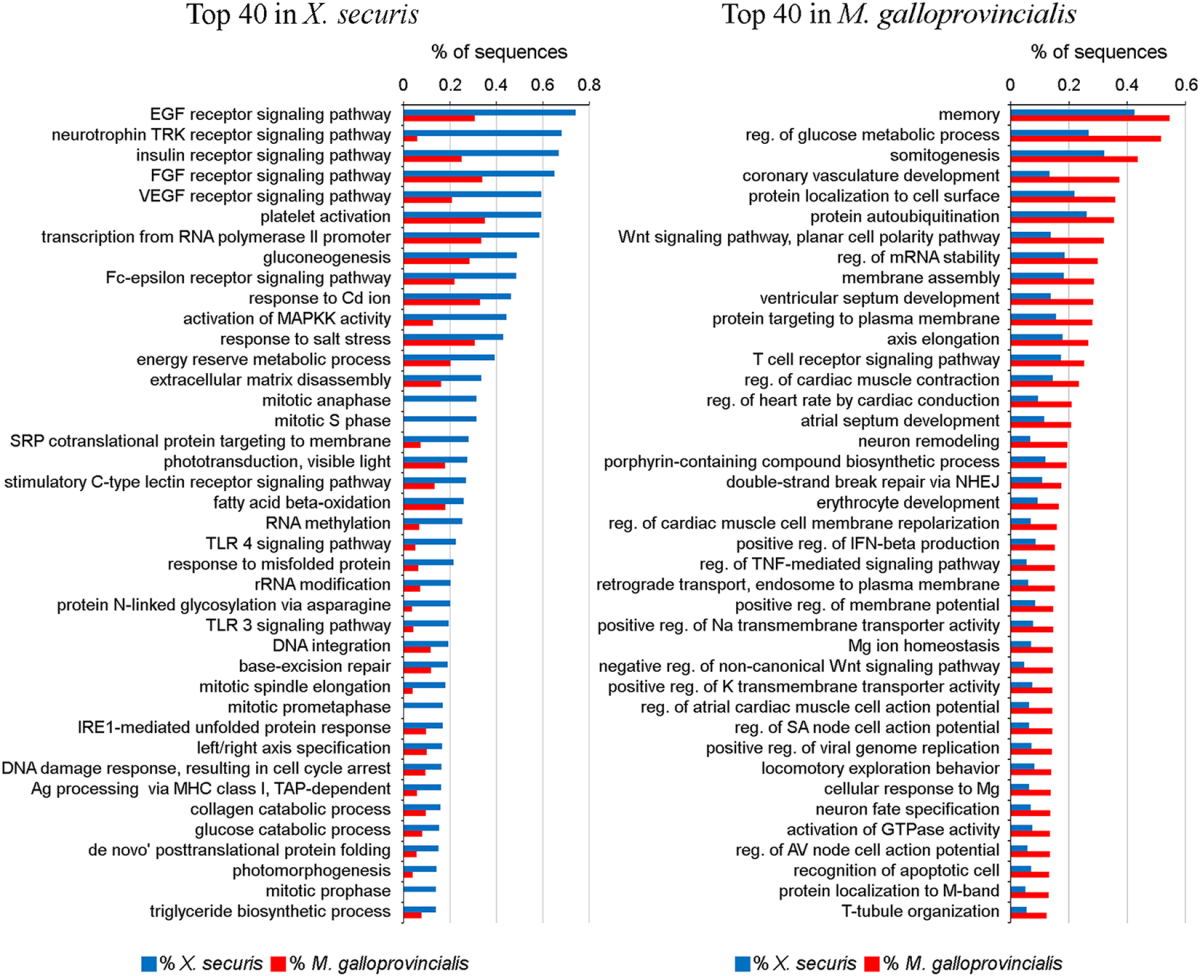
The 40 most significantly enriched biological processes in the transcriptomes of *X. securis* and *M. galloprovincialis*. Numbers indicate the percentage of sequences included in each category.

The RNA sequencing of mantle generated 123,478 million reads for X*. securis*, and 111,322 million reads were used for *M. galloprovincialis*. Those reads were assembled into 115,804 and 114,614 contigs for *X. securis* and *M. galloprovincialis*, respectively. The percentage of annotated contigs was 24.8% (28,751) for X*. securis* and 26.3% (30,200) for *M. galloprovincialis*. A list of contigs of *M. galloprovincialis* and *X. securis* including sequence name, length, subject mapping, e-value, GO, and TPM is supplied in the supplementary table 1. Transcriptomic differences were observed in the enrichment analyses (Fig. 2). The transcriptome of *X. securis* was significantly enriched in 134 processes, and 75% of the 40 most significant processes were involved in 3 main physiological functions: osmoregulation (e.g., response to salt stress), metabolism (e.g., glucose catabolic process and fatty acid beta-oxidation), and regulation of the cell cycle (e.g., G1 phase, S phase, prophase, prometaphase and anaphase). Signaling pathways related to epidermal, fibroblast and endothelial vascular growth factors or to mitogen-activated protein kinase kinase activity were also enriched in this animal (Fig. 2). In contrast, 184 enriched processes were identified in the transcriptome of *M. galloprovincialis*, but osmoregulation (e.g., positive regulation of Na transmembrane transporter activity and Mg ion homeostasis) and regulation of metabolism (e.g., regulation of glucose metabolic process) only represented 17.5% of the 40 most significant processes. Other relevant processes were the regulation of cardiac activity and several immune-related processes (Fig. 2). The complete list of all enriched processes is presented in Supplementary Table 2.

## Physiological tolerance to salinity stress

**Figure 3:**
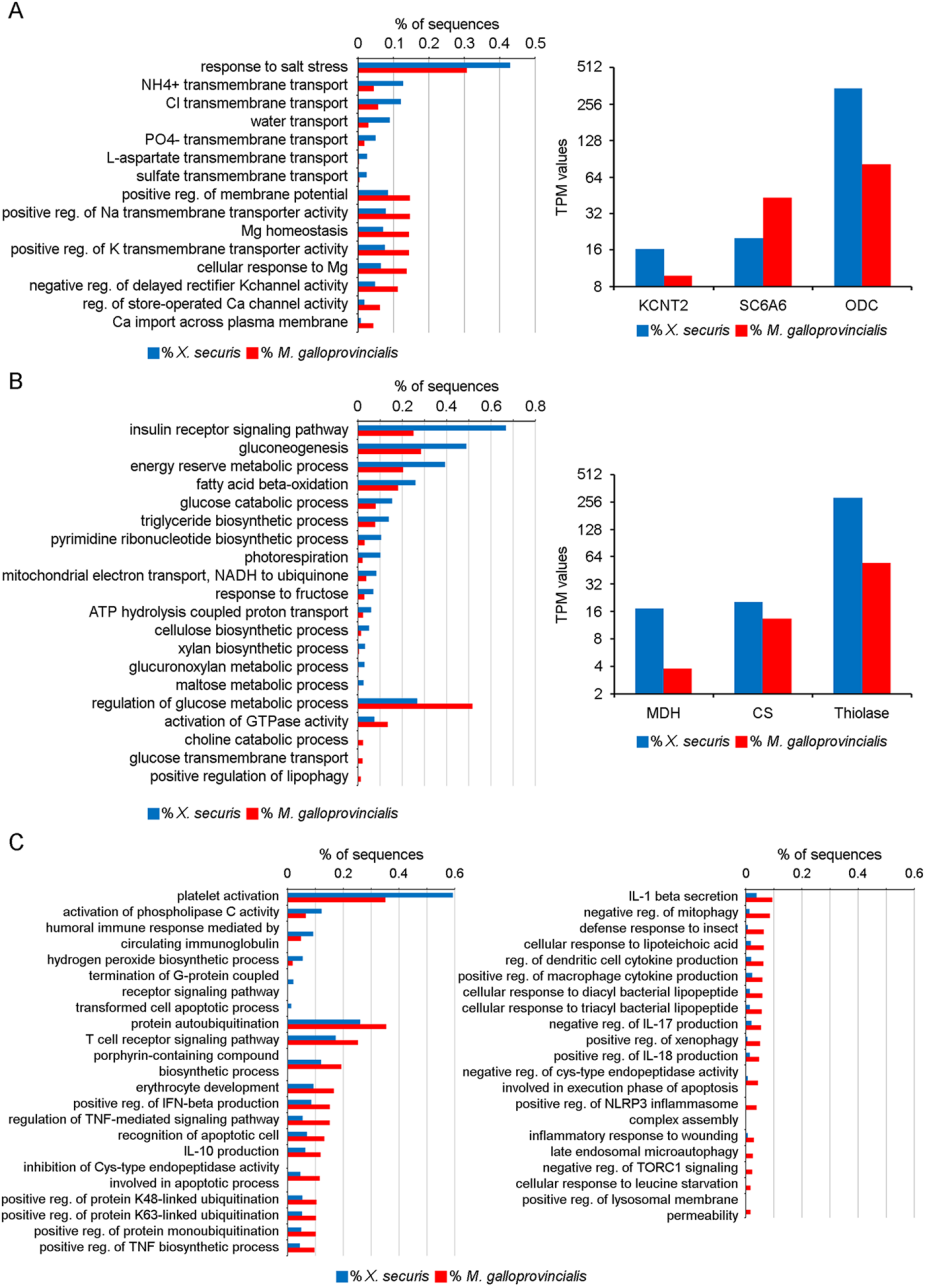
(**A**) GO terms related to osmoregulation (left). Expression levels (TPM) of key genes involved in osmoregulation and response to salinity stress (right). KCNT: K channel subfamily T gene. SCGA6: Taurine transporter gene. ODC: Ornithine decarboxylase gene. (**B**) GO terms related to metabolic processes (left). TPM values of genes involved in metabolism. Malate dehydrogenase (MDH), citrate synthase (CS) and thiolase genes. (**C**) Significantly enriched processes related to the immune response in the transcriptome of *X. securis* and *M. galloprovincialis*.

The transcriptomes of *X. securis* and *M. galloprovincialis* were enriched in general osmoregulatory processes such as positive regulation of ion transport (Na^+^, K^+^ or Mg^+2^), positive regulation of membrane potential and water transport. Interestingly, the GO term “response to salt stress” was the most-enriched process in *X. securis* (Fig. 3A). The basal expression levels of key genes involved in osmoregulation and in response to osmotic stress were analyzed. The K channel subfamily T gene (KCNT2), the taurine transporter gene SCGA6 and the ornithine decarboxylase (ODC) genes were selected. In our analysis, *X. securis* showed higher expression levels of the K channel (16.38 vs. 9.83) and the ODC gene (342.18 vs. 81.7) than *M. galloprovincialis*. Additionally, lower expression of the taurine transporter gene SCGA6 (19.95 vs. 43.5) in *X. securis* compared to *M. galloprovincialis* was observed (Fig. 3A).

### Metabolic cost of invasiveness

The transcriptome of *X. securis* was enriched in several metabolic pathways related to the use of sugars (gluconeogenesis, glucose catabolic processes, fructose and maltose metabolic processes) and lipids (fatty acid beta-oxidation and triglyceride metabolic processes). In contrast, only the metabolism of glucose was enriched in the transcriptome of *M. galloprovincialis* (Fig. 3B). The activation of the different metabolic pathways in *X. securis* was also supported by the higher basal expression level of key metabolic genes, such as malate dehydrogenase (MDH), citrate synthase (CS) and thiolase. TPM values for MDH and thiolase were 4.5 and 5 times higher, respectively, in *X. securis* than in *M. galloprovincialis* (17.3 vs. 3.8 for MDH and 281.65 vs. 54.7 for thiolase) (Fig. 3B).

### Immunological status to colonize new environments

The transcriptome of *X. securis* showed a lower number of enriched immune processes than those presented in *M. galloprovincialis* (Fig. 3C). In the Mediterranean mussel, specific responses, such as the regulation of the production of IL-1, IL-10, IL-17, IL-18, TNF, and IFN-beta, the cellular response to insects, the response to bacterial lipopeptides and the inflammatory response to wounding, were observed (Fig. 3C).

**Figure 4:**
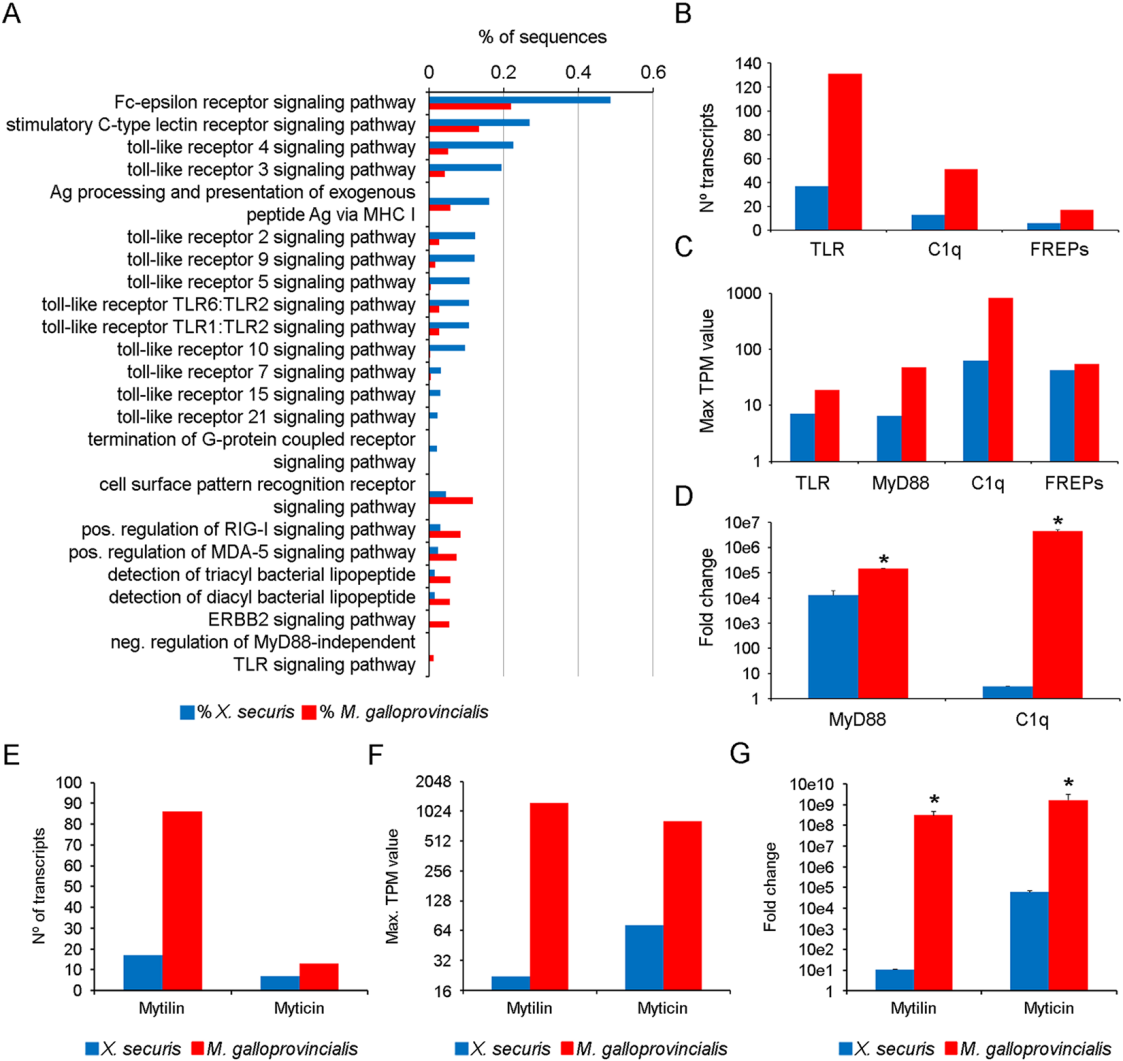
(**A**) Enriched processes related to receptors of pathogens in the transcriptome of *X. securis* and *M. galloprovincialis*. (**B**) Number of transcripts annotated as TLR, MyD88, FREPs and C1q. (**C**) Maximum expression values of the TLR, MyD88, FREPs and C1q genes. (**D**) Basal expression of MyD88 and C1q genes analyzed by qPCR. (**E**) Number of transcripts annotated as mytilins and myticins. **(F)** Maximum TPM value of the mytilin and myticin genes obtained in the RNA-seq. **(G)** Basal expression of mytilin and myticin genes by qPCR. (*)Significant differences at P < 0.0001.

The activation of the immune system is initiated by the recognition of pathogens through several receptor pathways. The transcriptome of *X. securis* was enriched in some of these pathways, such as the Toll-like receptor (TLR) pathway (TLRs 1, 2, 3, 4, 5, 6, 7, 9, 10, 15 and 21), the C-type lectin receptor pathway and the G protein-coupled receptor pathway (Fig. 4A). Fewer receptor pathways were found in the transcriptome of *M. galloprovincialis*: positive regulation of the RIG-1 and MDA-5 signaling pathway and two pathways for the detection of bacterial lipopeptides (Fig. 4A). However, when we analyzed the numbers of transcripts annotated as putative receptors (TLRs, fibrinogen-containing proteins (FREPs) and C1q-containing proteins) in the transcriptome, we found that they were 3 times higher in the transcriptome of *M. galloprovincialis* than in that of *X. securis* (Fig. 4B). The transcriptome of *M. galloprovincialis* included 131, 51 and 17 transcripts annotated as TLRs, C1q-containing proteins and FREPs, respectively, while only 37, 13 and 6 transcripts (TLR, C1q and FREPs, respectively) were present in *X. securis* (Fig. 4B). The maximum expression values (TPM) of those transcripts were also lower in *X. securis* than in *M. galloprovincialis*. In the case of TLR transcripts, the maximum expression was 2.5 times lower in *X. securis* (7 vs. 18.8), and expression of the TLR adaptor molecule MyD88 was 7.5 times lower in this invasive mussel (6.46 vs. 48.23). The highest difference in the expression values was observed for the C1q transcripts (62.42 vs. 831) (Fig. 4C). This lower expression of MyD88 and C1q genes was also confirmed by qPCR experiments using a new set of samples, which showed statistically significant differences between species (Fig. 4D).

We analyzed the repertoire of immune effector genes in the transcriptomes by measuring the number and expression of transcripts annotated as myticin and mytilin (Fig. 4E–G). Regarding mytilins, the number of transcripts and the maximum expression value were significantly lower in *X. securis* than in *M. galloprovincialis*. The alien *X. securis* presented one-fifth as many sequences annotated as mytilin compared to the autochthonous mussel (17 vs. 86) (Fig. 4E). Moreover, the highest TPM value of this gene in *X. securis* was 56 times lower than in *M. galloprovincialis* (21.95 vs. 1232.88) (Fig. 4F). This difference in gene expression between species was validated by qPCR in other samples with even higher values that reached differences of up to 10e7 (Fig. 4G). The analysis of myticin showed similar results. The autochthonous mussel presented 13 myticin transcripts, while only 7 transcripts were recorded in *X. securis* (Fig. 4E). Additionally, the maximum TPM value was 11 times lower in *X. securis* (73 vs. 810) (Fig. 4F), and the differences were significantly higher as shown in qPCR assays (up to 10e5) (Fig. 4G).

### Functional characterization of hemocytes of *X. securis*

**Figure 5:**
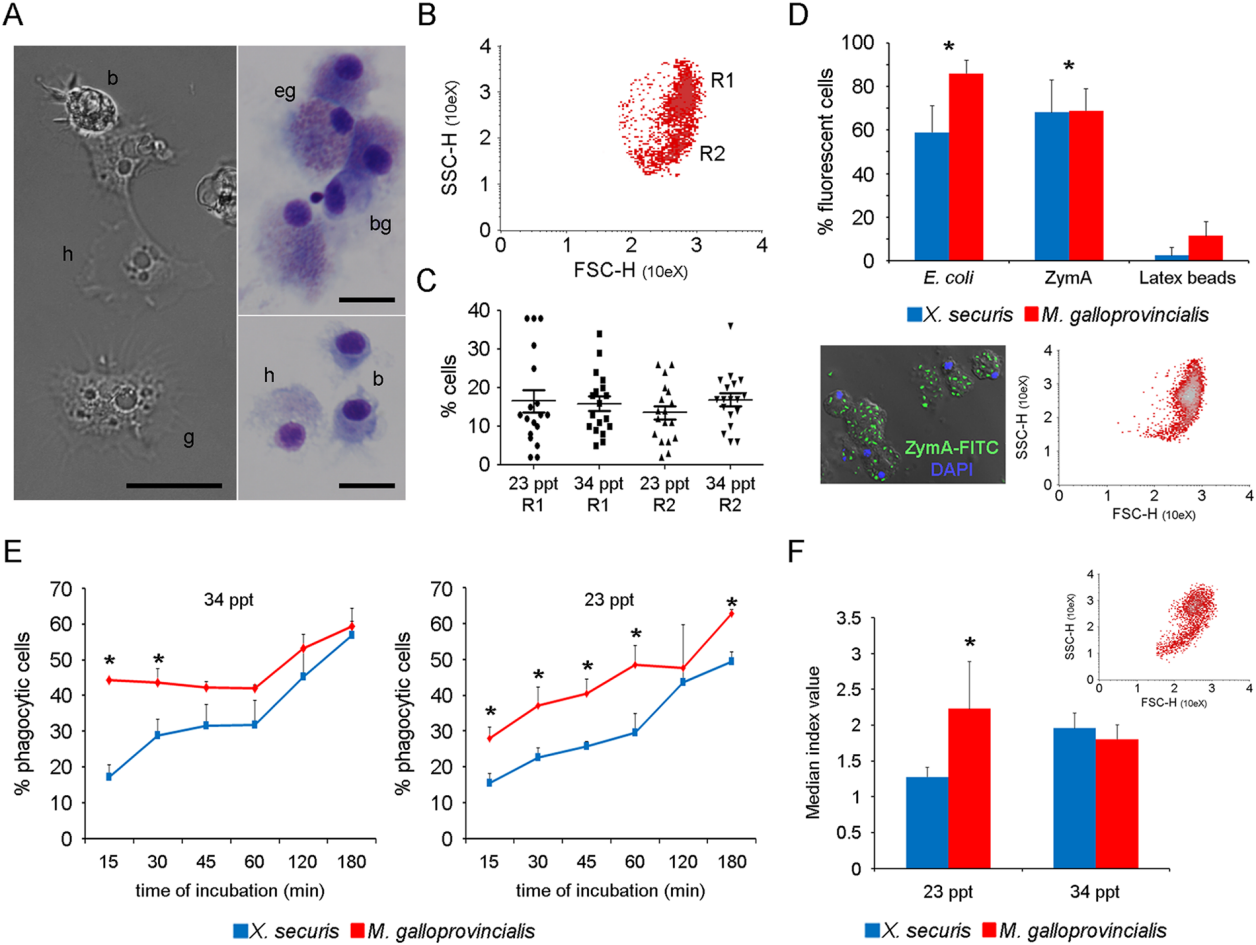
(**A**) Granulocytes (g), hyalinocytes (h) and blast-like cells (b) were observed. Eosinophilic (eg) and basophilic (bg) granulocytes were stained in pink and blue, respectively. Hyalinocytes (h) did not show colored granules in the cytoplasm. Scale bar 10 μm. (**B**) Representative dot-plots of cell size and granularity showing the distribution of hemocytes in *X. securis* maintained in sea water at 23 ppt salinity. Granulocytes and hyalinocytes were mainly included in R1 and R2, respectively. **(C)** Effect of water salinity on the number of hemocytes in each population (**D**) Percentage of cells engulfing particles after 3 h of incubation. (*)Significant differences at p < 0.005 compared to the group treated with latex beads. **(Inserts in D)** Image of *X. securis* hemocytes engulfing up to 15 ZymA-FITC particles. Phagocytic cells were mainly located in the R1 region. **(E)** Effect of salinity on the phagocytic activity and kinetics of hemocytes stimulated with ZymA-FITC particles. **(F)** Effect of water salinity on ROS production after stimulation with ZymA. Graphs in E and F represent the mean and SD of 3 independent experiments. (*)significant differences at p < 0.005. **(Insert in F)** ROS producer cells were mainly located in the R1 region.

Three cell types of circulating hemocytes were observed in *X. securis* defined as blast-like cells, hyalinocytes and granulocytes (Fig. 5A). Stained *X. securis* hemolymph smears showed pink eosinophilic granulocytes and blue basophilic granulocytes. Hyalinocytes did not show colored granules in the cytoplasm (Fig. 5A). Flow cytometric analysis only resolved two distinct subpopulations (granulocytes and hyalinocytes) (Fig. 5B). The acclimation of the animals to different sea water salinities (23 and 34 ppt) did not induce any significant variation in the number, size or complexity of the hemocyte populations (Fig. 5C).

We compared the capability of the hemocytes of both mussels to engulf different particles (latex beads, *E. coli* or ZymA). In both species, the percentages of cells engulfing *E. coli* and ZymA particles were significantly higher than those ingesting latex beads (Fig. 5D). No significant differences in phagocytosis were observed between *X. securis* and *M. galloprovincialis* after 3 h of incubation. Interestingly, significant differences were recorded at earlier times and when the water salinity was considered (Fig. 5E). The percentage of cells ingesting ZymA-FITC particles in *X. securis* was always lower than that registered in *M. galloprovincialis* at salinity 23 ppt but also at 34 ppt (Fig. 5E). In both species, the number of phagocytic cells increased over time. At 34 ppt, significant differences were observed only after 15 and 30 min of incubation. When the assay was conducted at 23 ppt, significant differences were obtained at almost all sampling points. At the end of the experiment, 62.6% (±0.3) of the *M. galloprovincialis* hemocytes ingested fluorescent particles, while only 49.4% (±0.8) of *X. securis* did so (Fig. 5E). Reactive oxygen species (ROS) were produced by hemocytes of both mussels after phagocytosis of ZymA, but significant differences were observed only at 23 ppt salinity (Fig. 5F). Cells producing ROS were mainly located in the R1 granulocyte region (insert in Fig. 5F).

### Susceptibility of *X. securis* to pathogens present in the ecosystem

**Figure 6:**
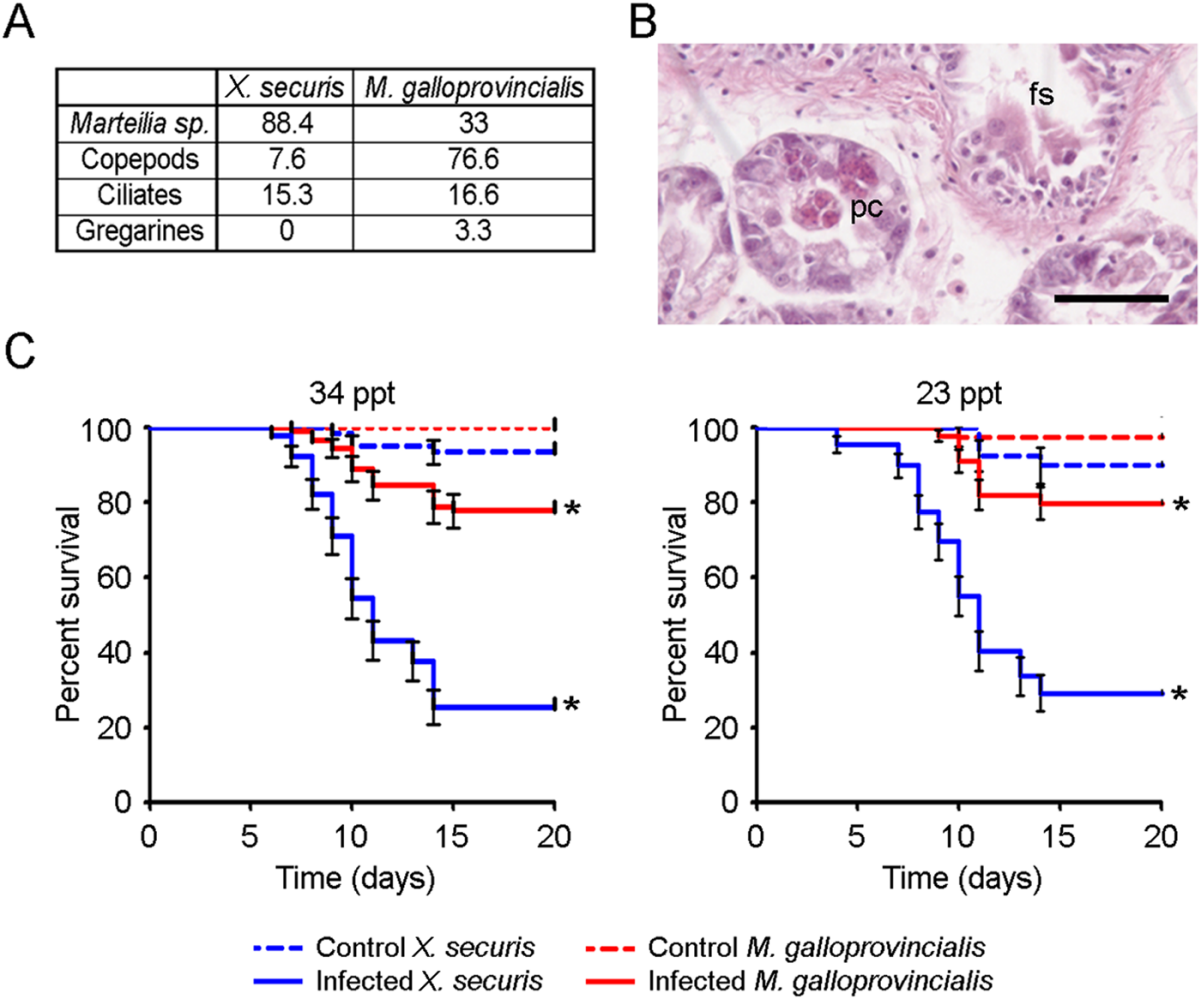
(**A**) Percentages of parasites in tissues from *X. securis* and *M. galloprovincialis* coexisting at the same location (intermediate sampling point). **(B)** Picture showing plasmodial cells (pc) in the epithelium of the digestive diverticula and final stages (fs) in the epithelium of the stomach. Scale bar: 50 μm. (**C**) Susceptibility of *X. securis* and *M. galloprovincialis* to *V. splendidus* LGP32 strain at native (23 ppt) and introduced (34 ppt) salinity ranges. (*) significant differences at P < 0.001.

To determine whether *X. securis* is less affected by enemies, we evaluated the diversity and abundance of parasites in *X. securis* and compared them with those of *M. galloprovincialis* coexisting at the same location. Our histopathological analysis revealed a lower prevalence of gregarines and copepods (e.g., *Mytilicola intestinalis*) in *X. securis* than in *M. galloprovincialis* (7.6% vs. 76.7%) but a higher prevalence of the protozoan *Marteilia* sp. (88.4% vs. 33%) (Fig. 6A). Plasmodial cells of the parasite were observed in the epithelium of the digestive diverticula and final stages in the epithelium of the digestive diverticula and stomach (Fig. 6B).

Experimental infections were conducted to evaluate the susceptibility of *X. securis* to the pathogenic bacterium *V. splendidus* LGP32. The percentages of survival were significantly lower in *X. securis* than in *M. galloprovincialis*, regardless of the water salinity (Fig. 6C). At 34 ppt, 76% of infected *M. galloprovincialis* survived the infection, while only 25% of *X. securis* were alive. Similar percentages were obtained at 23 ppt, at which 80 and 30% of *M. galloprovincialis* and *X. securis*, respectively, survived the infection (Fig. 6C).

## Discussion

The transcriptome of mantle from *X. securis* was compared with that of *M. galloprovincialis*. Although both transcriptomes were not performed at the same time and on individuals collected in the same place, this analysis was used as an exploratory tool for identifying broad differences between species in relevant physiological processes that allow *X. securis* to survive in and colonize the brackish ecosystem of the inner part of the Ría de Vigo. The percentages of annotated transcripts were similar in the two species and were consistent with other analyses conducted in *M. galloprovincialis*^25^ and *L. fortunei*^15^.

The top 40 biological processes presented in the enriched transcriptomes provide an overall image of the most important physiological processes that are activated in response to the environmental conditions. Osmoregulation is the most important process in *X. securis*, suggesting that daily variations in water salinity in the inner Ría induce physiological stress in this species. Salinity stress affects other processes, such as the cell cycle and metabolism^32^, which are also enriched in the transcriptome. In contrast, osmoregulation and metabolic processes only represented a small proportion (17%) of all the physiological processes in *M. galloprovincialis*, suggesting the absence of environmental stress in the outer part of the Ría and allowing the activation of other important processes such as the regulation of the immune system.

*X. securis* presented a limited number of enriched immune processes in contrast to the autochthonous *M. galloprovincialis*, suggesting a lower basal activity of the immune system in the invasive *X. securis* under natural conditions. This reduced innate immune response has also been described in other invasive animals, such as the wasp *Polistes dominulus*^33^, the house sparrow *Passer domesticus*^34^ and the cane toad *Rhinella marina*^35^, compared to the autochthonous species. This low basal immune activity of *X. securis* could be explained by the hypothesis proposed by Wilson-Rich and Starks^33^, who suggested that the immune response is dependent upon environmental conditions and a decrease in this response could be expected when it represents a waste of energetic resources.

The recognition of pathogens is the first step in the activation of the immune system. In mollusks, TLRs, FREPs and C1q are the main recognition factors^36–38^. The transcriptome of *X. securis* was enriched in several TLR pathways, but the low expression of the receptors and the MyD88 molecule suggests an upregulation of genes downstream of MyD88, which could result in a defective immune response with detrimental effects on its fitness^39^. In contrast, the lack of TLR pathways in the enriched transcriptome of *M. galloprovincialis* and the high expression of immune receptors (TLRs, FREPs and C1q) and MyD88 could suggest a fine regulation of the recognition processes to control the activation and the magnitude of the immune response.

Antimicrobial peptides are one of the most important classes of effector molecules in the immune system of mollusks^40^. They play a crucial role in the defense against Gram-positive and Gram-negative bacteria, viruses, fungi and parasites^41^. In our analysis, *X. securis* showed lower diversity and gene expression of myticin and mytilin compared to that presented in *M. galloprovincialis*. The elevated numbers of mytilin and myticin transcripts reflect the great expansion and diversity of those molecules in *M. galloprovincialis*, as previously reported^42,43^. The relationship between the AMPs and the resistance to pathogens in *X. securis* is worth exploration, since it could affect the dissemination of *X. securis* and its colonization of other neighboring locations. In *M. galloprovincialis*, the presence of a high number of mytilins and myticins, seems to be responsible for the low susceptibility to viral infections^44^.

Since a lower immune response of *X. securis* was suggested in the transcriptomic analysis, we demonstrate this difference at functional level. Bivalve hemocytes are immunocompetent cells that are able to mount a rapid and robust immune response against potential pathogens^45^. Three cell types of circulating hemocytes (blast-like cells, hyalinocytes and granulocytes) with morphologies and sizes similar to those described in other marine bivalves were observed in *X. securis*^46,47^. After activation, hemocytes participate in several pathogen-killing immune reactions, such as phagocytosis and ROS production^41^. Cells involved in the phagocytic activity and production of ROS were mainly granulocytes, in agreement with previous publications^41^. *X. securis* hemocytes showed a preference for *E. coli* and Zymosan particles, similar to hemocytes from other bivalve mollusks^47^ and echinoderms^48^. Phagocytosis and ROS production were always lower in *X. securis* than in *M. galloprovincialis*, suggesting that the magnitude of the immune response and the activity of the *X. securis* hemocytes are lower than those of *M. galloprovincialis*. Moreover, variations in water salinity could modulate the immune response of *X. securis*, as has been described in other marine invertebrate species^14^.

The transcriptomic assays and functional experiments highlighted the limited immune strength in *X. securis*. The ERH explains this reduction in immune capabilities because the alien species is less affected by enemies (pathogens or parasites) in its new ecosystem^8^. To evaluate this hypothesis, we analyzed the diversity and abundance of parasites in *X. securis* and compared them with those in *M. galloprovincialis* coexisting at the same location. Although *X. securis* seemed to be less vulnerable to predation, such as by *Carcinus maenas* or *Nucella lapillus*^49,50^, and could host fewer parasites than *M. galloprovincialis*^51^, our histopathological analysis revealed a higher prevalence of the protozoan *Marteilia* sp., which is a notifiable disease to the OIE and the EU, being one of the most serious causes of shellfish mortality worldwide^52^. Interestingly, *X. securis* in their native Australian habitat are not infected by *M. refringens* and *M. sydneyi*^53^. The high prevalence of *Marteilia sp*. in our new habitat could contribute to an increase in the prevalence of this disease in cultured mussels *M. galloprovincialis*, as suggested by Pascual *et al*.^51^. With all these results the ERH seems not to be completely responsible for the lower immune response of *X. securis*.

The relocation of energetic resources from immune mechanisms to other physiological processes necessary for survival could be another plausible explanation. However, one important consequence of this possibility is that invasive species could be more vulnerable to infections or novel parasites^10^. The consequences of the rearrangement of immune energetic resources were empirically confirmed, as the survival rate of *X. securis* was always lower than that of *M. galloprovincialis*, regardless of the water salinity. The high resistance of *M. galloprovincialis* to bacterial and viral infections has already been described^24,44^. It also could be possible that other factors besides rearrangement of energetic resources (e.g. prior exposure to Vibrio, genetic differences in susceptibility between the species) were involved in the differences in survival rates.

Immune response is costly in terms of energy resources, and a balanced and coordinated response with other physiological processes is essential to ensure the survival of the species^54^. It seems clear that osmoregulatory and metabolic processes are enriched in *X. securis*, to the detriment of immune capabilities. It is likely that the daily variations in salinity in the inner part of the Ría de Vigo induce a high physiological stress for this species, as can be suggested by the high expression of ion and amino acid transporters, usually modulated during a decrease in water salinity^55,56^. In particular, the taurine concentration is one of the major contributors to the acclimation to salinity changes in *Crassostrea gigas*^57^ and *Mytilus galloprovincialis*^58^. In bivalves, salinity stress can also affect the cell cycle^32,59^. Processes related to the regulation of the cell cycle were also enriched in the transcriptome of *X. securis*.

In aquatic animals, adaptation to the environmental conditions (e.g., water salinity, depth or temperature) represents a significant energy cost^60^. For example, it was estimated that the metabolic cost to maintain the ionic balance of sea urchin larvae represented up to 40% of the metabolic rate^61^. The demand for energy to cope with salinity stress results in the mobilization of carbohydrates, lipids and even protein reserves^62,63^, as can be inferred in the *X. securis* transcriptome in contrast to that of *M. galloprovincialis*. This observation was confirmed by the evaluation of the basal expression of key metabolic genes, such as malate dehydrogenase (MDH), citrate synthase (CS), and thiolase. Variations in MDH have been reported in lobsters and polychaete annelids in association with salinity^64,65^. *X. securis* showed higher basal expression values of all those enzymes compared to *M. galloprovincialis*, suggesting a higher basal demand to obtain the energy for physiological processes.

In conclusion, our results suggest that the introduction of *X. securis* to this new environment could impose high energetic costs that resulted in the mobilization of sugars and lipids as energy reserves for the activation of osmoregulatory processes to cope with salinity stress. In this scenario, the reallocation of energetic resources from the immune response to vital physiological processes could be a reasonable explanation for the low immune capabilities observed in *X. securis*. It is difficult to predict the potential invasiveness of *X. securis* and the hypothetical replacement of the native mussel. *M. galloprovincialis*, with a stronger immune response, could continue to be the dominant mussel species in the outer part of the Ría de Vigo, but *X. securis*, with stronger constitutive physiological responses, could spread if the physiological and metabolic requirements do not affect its immune capabilities.

The application of integrated transcriptomic and functional immunological approaches is useful to understand the biology of an invasive species. Therefore, integrated eco-immunological analysis is an extremely promising area of research whose findings could be used to guide and inform management decisions.

### Data accessibility

The raw data for *X. securis* are accessible from the NCBI database under Bioproject ID: PRJNA470586 and Short Read Archive ID: SRP145061. The raw data of the RNA sequencing of the mantle samples from *M. galloprovincialis* were downloaded from Moreira *et al*.^25^.

## Supplementary information

Supplementary figure 1

Supplementary table 1

Supplementary table 2

Supplementary table 3

Supplementary dataset 1

Supplementary dataset 2

## References

[1] Early R (2016). Global threats from invasive alien species in the twenty-first century and national response capacities. Nat. Commun..

[2] Pimental, D. Environmental consequences and economic costs of alien species in Invasive Plants: Ecological and Agricultural Aspects (eds. Inderjit.) 269–276 (Birkhäuser Verlag/Switzerland 2005).

[3] Lowry E (2013). Biological invasions: a field synopsis, systematic review, and database of the literature. Ecol. Evol..

[4] Ruiz GM, Fofonoff PW, Carlton JT, Wonham MJ, Hines AH (2000). Invasion of coastal marine communities in North America: apparent patterns, processes, and biases. Annu. Rev. Ecol. Syst..

[5] McKindsey CW, Landry T, O’Beirn FX, Davies AM (2007). Bivalve aquaculture and exotic species: review of ecological considerations and management issues. J. Shellfish Res..

[6] Chan FT, Briski E (2017). An overview of recent research in marine biological invasions. Marine Biol..

[7] Sadd BM, Schmid-Hempel P (2008). Principles of ecological immunology. Evol. Applications.

[8] Colautti RI, Ricciardi A, Grigorovich IA, MacIsaac HJ (2004). Is invasion success explained by the enemy release hypothesis?. Ecology Letters.

[9] Cornet S, Brouat C, Diagne C, Charbonnel N (2016). Eco-immunology and bioinvasion: revisiting the evolution of increased competitive ability hypotheses. Evolutionary Applications.

[10] White TA, Perkins SE (2012). Invasions and Infections. The ecoimmunology of invasive species. Functional Ecol..

[11] Vogel H, Schmidtberg H, Vilcinskas A (2017). Comparative transcriptomics in three ladybird species supports a role for immunity in invasion biology. Dev. Comp. Immunol..

[12] Rius M, Bourne S, Hornsby HG, Chapman MA (2015). Applications of next-generation sequencing to the study of biological invasions. Current Zoology.

[13] Sherman CDH (2016). What are we missing about marine invasions? Filling in the gaps with evolutionary genomics. Marine Biol..

[14] Lockwood BL, Connor KM, Gracey AY (2015). The environmentally tuned transcriptomes of Mytilus mussels. J. Exp. Biol..

[15] Uliano-Silva M (2014). Gene Discovery through Transcriptome Sequencing for the Invasive Mussel Limnoperna fortune. PLoS One.

[16] Verbruggen B (2015). De novo assembly of the Carcinus maenas transcriptome and characterization of innate immune system pathways. BMC Genomics.

[17] Vilcinskas A, Mukherjee K, Vogel H (2013). Expansion of the antimicrobial peptide repertoire in the invasive ladybird Harmonia axyridis. Proc. Royal Soc. London B.

[18] Garci ME (2007). Xenostrobus securis (Lamarck, 1819) (Mollusca: Bivalvia): first report of an introduced species in Galician waters. Aqua. Int..

[19] Morton B, Leung KF (2015). Introduction of the alien Xenostrobus securis (Bivalvia: Mytilidae) into Hong Kong, China: Interactions with and impacts upon native species and the earlier introduced Mytilopsis sallei (Bivalvia: Dreissenidae). Mar. Poll. Bull..

[20] Barbieri M (2011). New records of the pygmy mussel Xenostrobus securis (Bivalvia: Mytilidae) in brackish-water biotopes of the western Mediterranean province: evidence of its invasive potential. Mar. Biodiversity Rec..

[21] Streftaris N, Zenetos A (2006). Alien marine species in the Mediterranean–the 100 ‘worst invasives’ and their impact. Mediterranean Mar. Sci..

[22] Guerra A (2013). The black-pygmy mussel Limnoperna securis in Galician Rias (north-eastern Atlantic): new records and first evidence of larval stages predation by copepods. Mar. Biodiversity Rec..

[23] Gestoso I (2013). Shifts from native to non-indigenous mussels: enhanced habitat complexity and its effects on faunal assemblages. Mar. Env. Res..

[24] Romero A (2014). Occurrence, seasonality and infectivity of Vibrio strains in natural populations of mussels Mytilus galloprovincialis. Dis. Aqua. Org..

[25] Moreira R (2015). RNA-Seq in Mytilus galloprovincialis: comparative transcriptomics and expression profiles among different tissues. BMC Genomics.

[26] Conesa A (2005). Blast2GO, a universal tool for annotation, visualization and analysis in functional genomics research. Bioinformatics.

[27] Madeira F (2019). The EMBL-EBI search and sequence analysis tools APIs in 2019. Nucleic Acids Res.

[28] Rozen, S. & Skaletsky, H. J. Primer3 on the WWW for general users and for biologist programmers in Bioinformatics Methods and Protocols: Methods in Molecular Biology (eds Krawetz, S., Misener, S.) 365–386 (Totowa, NJ: Humana Press, 2000). 10.1385/1-59259-192-2:365 10547847

[29] Pfaffl MW (2001). A new mathematical model for relative quantification in real-time RT-PCR. Nucleic Acids Res..

[30] Howard, A. W. & Smith, C. S. Histological techniques for marine bivalve mollusks. NOAA Technical Memorandum NMFS-F/NEC-25, Woods Hole (1983).

[31] Balbi T (2013). Interactions between Mytilus galloprovincialis hemocytes and the bivalve pathogens Vibrio aestuarianus 01/032 and Vibrio splendidus LGP32. Fish Shellfish Immunol..

[32] Zhao X, Yu H, Kong L, Li Q (2012). Transcriptomic Responses to Salinity Stress in the Pacific Oyster Crassostrea gigas. PLoS One.

[33] Wilson-Rich N, Starks PT (2010). The Polistes war: weak immune function in the invasive Polistes dominulus relative to the native P. fuscatus. Insectes Sociaux.

[34] Lee KA, Martin LB, Wikelski M (2005). Responding to inflammatory challenges is less costly for a successful avian invader, the house sparrow (Passer domesticus), than its less-invasive congener. Oecologia.

[35] Llewellyn D, Thompson MB, Brown GP, Phillips BL, Shine R (2012). Reduced investment in immune function in invasion-front populations of the cane toad (Rhinella marina) in Australia. Biol. Invasions.

[36] Coscia MR, Giacomelli S, Oreste U (2011). Toll-like receptors: an overview from invertebrates to vertebrates. Invertebrate Survival J..

[37] Jiang S (2015). A C1q domain containing protein from Crassostrea gigas serves as pattern recognition receptor and opsonin with high binding affinity to LPS. Fish Shellfish Immunol..

[38] Romero A (2011). Individual sequence variability and functional activities of fibrinogen-related proteins (FREPs) in the Mediterranean mussel (Mytilus galloprovincialis) suggest ancient and complex immune recognition models in invertebrates. Dev. Comp. Immunol..

[39] Sears BF, Rohr JR, Allen JE, Martin LB (2011). The economy of inflammation: when is less more?. Trends in Parasitol..

[40] Zannella C (2017). Microbial Diseases of Bivalve Mollusks: Infections, Immunology and Antimicrobial Defense. Mar. Drugs.

[41] Allam B, Raftos D (2015). Immune responses to infectious diseases in bivalves. J. Invertebrate Pathol..

[42] Costa MM (2009). Evidence of high individual diversity on myticin C in mussel (Mytilus galloprovincialis). Dev. Comp. Immunol..

[43] Mitta G, Vandenbulcke F, Hubert F, Salzet M, Roch P (2000). Involvement of mytilins in mussel antimicrobial defense. J. Biol. Chemistry.

[44] Novoa B (2016). Antiviral Activity of Myticin C Peptide from Mussel: an Ancient Defense against Herpesviruses. J. Virol..

[45] Hine PM (1999). The inter-relationships of bivalve haemocytes. Fish Shellfish Immunol..

[46] Donaghy L, Kim BK, Hong HK, Park HS, Choi KS (2009). Flow cytometry studies on the populations and immune parameters of the hemocytes of the Suminoe oyster Crassostrea ariakensis. Fish Shellfish Immunol..

[47] Prado-Alvarez M (2012). Morphological characterization and functional immune response of the carpet shell clam (Ruditapes decussatus) haemocytes after bacterial stimulation. Fish Shellfish Immunol..

[48] Romero A, Novoa B, Figueras A (2016). Cell mediated immune response of the Mediterranean sea urchin Paracentrotus lividus after PAMPs stimulation. Dev. Comp. Immunol..

[49] Babarro JMF, Vázquez E, Olabarria C (2016). Importance of phenotypic plastic traits on invasion success: response of Xenostrobus securis to the predatory dogwhelk Nucella lapillus. Mar. Ecol. Progress Series.

[50] Veiga P (2011). Does Carcinus maenas facilitate the invasion of Xenostrobus securis?. J. Exp. Mar. Biol. Ecol..

[51] Pascual S (2010). The mussel Xenostrobus securis: a well-established alien invader in the Ria de Vigo (Spain, NE Atlantic). Biological Invasions.

[52] Aranguren, R., Poisa-Beiro, L., Villalba, A. & Figueras, A. Marteiliosis en moluscos in Enfermedades de moluscos bivalvos de interés en acuicultura (eds. A. Figueras, A. & Novoa B.) 243–272 (Publicaciones científicas y tecnológicas de la fundación OESA, Madrid, Spain, 2011).

[53] Colgan, D. J. The Australian Museum. *Technical Report for the NSW Environmental Trust Seeding Grant* 2012/RDS/002 (2014).

[54] Zuk M, Stoehr AM (2002). Immune defense and host life history. American Naturalist.

[55] Lin CH, Yeh PL, Lee TH (2016). Ionic and Amino Acid Regulation in Hard Clam (Meretrix lusoria) in Response to Salinity Challenges. Frontiers in Physiol..

[56] Pierce SK (1982). Invertebrate cell volume control mechanisms: a coordinated use of intracellular amino acids and inorganic ions as osmotic solute. Biol. Bull..

[57] Lee N, Han K, Choi K (2004). Effects of salinity and turbidity on the free amino acid composition in gill tissue of the pacific oyster, Crassostrea gigas. J. Shellfish Res..

[58] Babarro JM, Fernández Reiriz MJ (2006). Variability of taurine concentrations in Mytilus galloprovincialis as a function of body size and specific tissue. Comp. Bioche. Physiol. - Part B: Biochem. Mol. Biol..

[59] Lockwood BL, Somero GN (2011). Transcriptomic responses to salinity stress in invasive and native blue mussels (genus Mytilus). Mol. Ecol..

[60] Seibel BA, Drazen JC (2007). The rate of metabolism in marine animals: environmental constraints, ecological demands and energetic opportunities. Philosophical Trans. Royal Soc. of London. Series B Biol. Sci..

[61] Leong PKK, Manahan DT (1997). Metabolic importance of Na+/K+-ATPase activity during sea urchin development. J. Exp. Biol..

[62] Martis TL (2011). Effects of hypo- or hyperosmotic stress on lipid synthesis and glucogenic activity in tissues of the crab Neohelice granulate. Comp. Biochem. Physiol. Part A.

[63] Telahigue K, Rabeh I, Chetoui I, Romdhane MS, El-Cafsi M (2010). Effects of decreasing salinity on total lipids and fatty acids of mantle and gills in the bivalve Flexopecten glaber (Linnaeus, 1758) under starvation. Cahiers De Biologie Marine.

[64] Cripps RA, Reish DJ (1973). The effect of environmental stress on the activity of malate dehydrogenase and lactate dehydrogenase in Neanthes arenaceodentata (annelida: Polychaeta). Comp. Biochem. Physiol..

[65] Gould E (1980). Low-salinity stress in the American lobster, Homarus americanus, after chronic sublethal exposure to cadmium: Biochemical effects. Helgoländer Meeresunters.

