# Supplementary material for: Integrated transcriptomic and functional immunological approach for assessing the invasiveness of bivalve alien species
Source: Sci Rep. 2019 Dec 27;9:19879. doi: 10.1038/s41598-019-56421-y (PMC6934813; doi:10.1038/s41598-019-56421-y)
Supplement: Supplementary file 1 — Supplementary figure 1 [file 41598_2019_56421_MOESM1_ESM.docx]

**Integrated transcriptomic and functional immunological approach for assessing the invasiveness of bivalve alien species.**

Alejandro Romero, Raquel Aranguren, Rebeca Moreira, Beatriz Novoa*, Antonio Figueras

**Supplementary figure 1**

Alignment of sequences from *X. securis* and *M. galloprovincialis* used for the analysis of gene expression by qPCR. The position of the primers was highlighted in the alignment. The sequences of forward primers are highlighted in blue. The sequences of reverse primers are highlighted in pink.

**C1q sequences used for qPCR experiments**

C1q64_Xenostrobus TTCACGTTATCCTAACCCTTCTGGAGAATATAATTACGGGGAATATGGTACAGAACTAAT 60

C1q64_Mytilus TTCACGTTATACAAATCCCCCTGGAAGATTAAACTACGGGGAGTATGGAACAGAATTAAT 60

********** * ** ** ***** ** ** ******** ***** ****** ****

C1q64_Xenostrobus GATGGGCAGTAGAAAAATCGGTGTTTTACATACAGACACTGAGAAAAGGTACGACGACGC 120

C1q64_Mytilus GATGGGCAGCACAAAAATCGGTGTTCTACATACAGATACAGAGACAAAGTACGACGACGC 120

********* * ************* ********** ** **** ** ************

C1q64_Xenostrobus ATGCTCCACAGGATTTGTCATCAAATATATACCACATAAGAATCAAGTTTATGTCAGAAA 180

C1q64_Mytilus ATGCTCCACTGGATTTGTCATCAGATATGTATCATCAGGCAATCAAGTTTACGTCAGAAA 180

********* ************* **** ** ** *********** ********

C1q64_Xenostrobus TACCTACGCTCACCAAGGTCGTCTGCTAAGCAGGGAAAGTCAGACCAGAACAACGTTTTC 240

C1q64_Mytilus TAACTACGCCCACCAAGGCAAACTTCTAAGCAAGGAAAGTCAGACCAGAACAACTTTCTC 240

** ****** ******** ** ******* ********************* ** **

C1q64_Xenostrobus GGGATGGAAAATGCAATAAAAGGC 264

C1q64_Mytilus TGGATGGAAAATGCAATAAAAAGG 264

******************** *

**Mytilin sequences used for qPCR experiments**

Mytilin_Xenostrobus GCAGTTATTCTGGCTATCGCTCTTGTAGCAATTCTTGCAGTCCATGAGGCAGAGGCAAGT 60

MytilinC_Mytilus GCAGTTATCCTAGCCATCGCCCTTGCAGTACTTCTTATAGTGCAAGACGCAGATGCAAGC 60

******** ** ** ***** **** ** * ***** *** ** ** ***** *****

Mytilin_Xenostrobus TGTGCTTCCAGATGTAAAGGCCATTGTAGAGCAAGACGCTGTGGATATTATGTATCAGTC 120

MytilinC_Mytilus TGTGCTTCCAGATGTAAATCTCGTTGTAGAGCCAGACGCTGTAGATATTACGTGTCAGTC 120

****************** * ********* ********* ******* ** ******

Mytilin_Xenostrobus CTATACAGAGGGCGTTGCTACTGCAAATGTCTTCGTTGTTCCAGTGAGCATTCCATGAAA 180

MytilinC_Mytilus AGATATGGATGGTTTTGCTATTGCAGATGTCTCCGTTGTTCCAGCGAGCATACCATGAAA 180

*** ** ** ****** **** ****** *********** ****** ********

Mytilin_Xenostrobus TTCCCTGAAAATGAAGGATCATCTCCATCTGACATGATGCCACAGATGAATGAAAATGAG 240

MytilinC_Mytilus TTCTCCCCTGAAAGTGAAGG---ACCAGCTGAGATGCCAGCACAGATGAATGACCATGAG 237

*** * * * * *** **** *** ************* *****

Mytilin_Xenostrobus AACACTGAATTCGGTCAGGACATGCCCACAGGAGAAACCGAACAAGGTGAAACTGGCATT 300

MytilinC_Mytilus C---------AATTCCAGGACATGCAGAAAGGAGAAACCGAACAAGGTGAAACTGGAATG 288

********** * *************************** **

**Myticin sequences used for qPCR experiments**

Myticin_Xenostrobus -------------------------------------------TTGGAGTTCAGGAAGCC 17

Myticin_Mytilus ATGAAGGCAACAATCTTGTTAGCAGTTCTAGTGGCAGTCTTTGTCGCAGGTACGGAAGCT 60

* * ** * ******

Myticin_Xenostrobus CAATCAGTAGCTTGTACATCATACTACTGTAGTAAGTTCTGTGGGTCTGCTGGTTGCTCA 77

Myticin_Mytilus CATTCGCACGCTTGTACATCATACTGGTGTGGTAAGTTTTGTGGAACTGCTAGTTGCACA 120

** ** **************** *** ******* ***** ***** ***** **

Myticin_Xenostrobus TTATATGGATGTTACCTACTTCATCCTGGCAAAATTTGCTACTGCCTTCATTGTCGCAGA 137

Myticin_Mytilus CATTATTTATGCAGAGTACTCCATCCCGGTAAACTGTGTGTATGTGTTCATTGCAGCAGG 180

*** *** **** ***** ** *** * ** ** ******* ****

Myticin_Xenostrobus GCTGAGTCTCCATTGGCACTTTCTGGAAGCGCTAGGAATGTGAACGACAAGAACAACGAG 197

Myticin_Mytilus GTGAACAATCCTTTCAGAGTTAATCAAGTTGCTAAAAGTAT------------TAACGAT 228

* * *** ** * ** * * **** * * * *****

Myticin_Xenostrobus ATGGACAACTCTCCAATGATGAATGAGGTGGAACATTTGGACCAAGAAATG--------- 248

Myticin_Mytilus TTGGATTACACTCCAATAATGAAGTCGATGGAAAACTTGGACAATGGAATGGATATGTTA 288

**** ** ******* ***** * ***** * ****** * * ****

**MyD88 sequences used for qPCR experiments**

MyD88b_M-galloprovincialis_JX112712.1 CCGAGTAGCCGCCACTATCAAGTGCCCTCTCGTCCCAAGTATACAGGATTGGC------- 1113

MYD88_Mytilus CCGAGTAGCCGCCACTATCAAGTGCCCTCTCGTCCCAAGTATACAGGATTGGC------- 891

MyD88b_M-galloprovincialis_KC357781.1 CAGACTAGCTAAGTCAGTGAAGGCACCATTAGATCCTGAACCTAATCGTATGGGCTCTAG 1165

MYD88_Xenostrobus AAGACTTGCCAAGTCTGTGAAAGCTCCTTTAGATCCAGATTCAGCTCGGCATGGGACCTC 995

** * ** * * ** ** * * **

MyD88b_M-galloprovincialis_JX112712.1 ------------------AGTGTACAA------------------TTGATGACTTAAAGA 1137

MYD88_Mytilus ------------------AGTGTACAA------------------TTGATGACTTAAAGA 915

MyD88b_M-galloprovincialis_KC357781.1 TGTAGAGTCATCTTCACCGATGTCCATACCTTCCAATTCTTCATCTTCTCTGCAGTCACA 1225

MYD88_Xenostrobus TGTAGAATCTTCATCTCCTATGTCAATTTCTTCATCGTCCTCTTCCTTGTCAAACTCA-- 1053

*** * * *

MyD88b_M-galloprovincialis_JX112712.1 ATATGAAATATGACAGTAAGCATGTGCAACATCTGTTGTATGGAATTGGTGTTGCATGTC 1197

MYD88_Mytilus ATATGAAATATGACAGTAAGCATGTGCAACATCTGTTGTATGGAATTGGTGTTGCATGTC 975

MyD88b_M-galloprovincialis_KC357781.1 AACCTCGCTTTCA---------CCATCACAATCTCTGTCGTCTATTGAAAATTACCGTCT 1276

MYD88_Xenostrobus -ACATCGATATCAATGTCCCCATCATCATCTTCTCTATCAGGAATCGGCTCGTACTGTTT 1112

* * * ** *** * * * *

MyD88b_M-galloprovincialis_JX112712.1 AAGAATTTCCACCCGAAGAAGAAGAAGAAAAGAAA---GGAAAGAAAGGAGAAAAGAAGA 1254

MYD88_Mytilus AAGAATTTCCACCCGAAGAAGAAGAAGAAGAAAAGAAAGGAAAGAAAGGAGAAAAGAAGA 1035

MyD88b_M-galloprovincialis_KC357781.1 TACAAATGACTCAA--TAAAGAATGCACAGCGTCAGCTATCAGAGTCAGCATCAATGTCA 1334

MYD88_Xenostrobus GACAAATGCCTCAA--TGAAAAACGCTCAGAAAATGTTGGAAGACACCGACCCATCTTCA 1170

* ** * * * ** ** * * * * *

MyD88b_M-galloprovincialis_JX112712.1 AGAAAACTTGATGAAGAAT-----------GTGTCAACGTATTTAGATTTAATTGA---A 1300

MYD88_Mytilus AGAAAACTTGA------------------------------------------------- 1046

MyD88b_M-galloprovincialis_KC357781.1 AGTAACACGAGTAGTGACTCTGGATTTCACTCTTCGTCATTCCAGTCTCAAGGGGCC--T 1392

MYD88_Xenostrobus ACCGAGCAAGATTCAGGATTTATATCACTTCCGTCAACAAGCCGGAGTCTCAGTAATGAT 1230

* *

MyD88b_M-galloprovincialis_JX112712.1 GATCGATGTTTGTCAGAAAAATCTGATACAG-----ACGT-------------------- 1335

MYD88_Mytilus ------------------------------------------------------------ 1046

MyD88b_M-galloprovincialis_KC357781.1 ---CGTCGCTTCAATCAAATCAAAGTAGCTTCAGTAACGAATATACATCATTATCTACAG 1449

MYD88_Xenostrobus GAGCCATCATTTTCCCAAGAATATGAATCTTTTGTGCCGGATATCAGGCATTCAC----- 1285

MyD88b_M-galloprovincialis_JX112712.1 ---GTATCCGTCCATTTATCACTTAGAGATCAT---TAAAGAAATCCAA----------- 1378

MYD88_Mytilus ------------------------------------------------------------ 1046

MyD88b_M-galloprovincialis_KC357781.1 AGAGTTTCCCTACTTCATCGAGTAAGGCACGAGAAAAAAAGAAGGGTACTAATATATTTG 1509

MYD88_Xenostrobus ---------CTCCTACTACACCATCAGTTCCAAGGCAAAAGAAGGACAAGGGCAAATCAA 1336

MyD88b_M-galloprovincialis_JX112712.1 -ACATC------CAAATGGATGTGTTATAACACTGTAGACCCATACAGGAACTGTCGACT 1431

MYD88_Mytilus ------------------------------------------------------------ 1046

MyD88b_M-galloprovincialis_KC357781.1 GACAATTAAAAGCAAAGGTATCTGGAAAGAAGCACAATAATAATACAAGTAGTCAAGC-- 1567

MYD88_Xenostrobus AAAATTTCATTACTAAAATATTTAGCAAGAAAAATAACACTCACACAAGTAGTCAAGC-- 1394

**EFa1 sequences used for qPCR experiments**

EFa1_Xenostrobus GACTCTATTCTTCCACCCTCAAGACCAACAGACAAAGCTCTCCGTCTCCCACTTCAGGAT 60

EFa1_Mytilus GATTCTATCCTCCCACCATCAAGACCCACAGACAAAGCTCTCCGTCTCCCACTCCAGGAT 60

** ***** ** ***** ******** ************************** ******

EFa1_Xenostrobus GTCCATAAAATAGGAGGTATTGGAACTGTCCCAGTAGGCAGAGTAGAAACTGGTATCATT 120

EFa1_Mytilus GTTTACAAAATTGGAGGTATTGGAACAGTGCCAGTAGGTAGAGTAGAAACTGGAATCATC 120

** * ***** ************** ** ******** ************** *****

EFa1_Xenostrobus AAGCCTGGTATGGTTGTTGTTTTTGCTCCACCAAACATCACAACTGAGGTCAAATCTGTA 180

EFa1_Mytilus AAACCAGGTATGGTTGTTACCTTTGCTCCAGCCAACATCAGTACTGAAGTAAAGTCCGTA 180

** ** ************ ********* * ******* ***** ** ** ** ***

EFa1_Xenostrobus GAAATGCACCACGAATCTTTACCTGAAGCTTTACCTGGTGACAATGTTGGTTTCAACATC 240

EFa1_Mytilus GAAATGCACCACGAGTCTCTCCCAGAAGCTTTACCAGGAGACAATGTTGGTTTCAACGTA 240

************** *** * ** *********** ** ****************** *

EFa1_Xenostrobus AAGAACGTCTCGGTCAAAGAAATCAAAAGAGGAAACGTATGTGGAGACAGCAAGAATGAT 300

EFa1_Mytilus AAGAACGTCTCTGTCAAGGAAATTCGTAGAGGAATGGTCTGTGGTGACAGCAAAAATGAC 300

*********** ***** ***** ******* ** ***** ******** *****

EFa1_Xenostrobus CCACCAAAAGGAGCCAAGAGCTTCCTTGCACAGGTCATCATCTTGAACC 349

EFa1_Mytilus CCACCCAAGGGAGCCAAAAGTTTTGTTGCACAGGTCATCATCTTGAACC 349

***** ** ******** ** ** ************************
